# English-Speaking Adults' Labeling of Child- and Adult-Directed Speech Across Languages and Its Relationship to Perception of Affect

**DOI:** 10.3389/fpsyg.2021.708887

**Published:** 2021-09-01

**Authors:** Melanie Soderstrom, Marisa Casillas, Megan Gornik, Alexis Bouchard, Sarah MacEwan, Anahita Shokrkon, John Bunce

**Affiliations:** ^1^Department of Psychology, University of Manitoba, Winnipeg, MB, Canada; ^2^Department of Comparative Human Development, University of Chicago, Chicago, IL, United States; ^3^Département d'Éducation, Université de Saint-Boniface, Winnipeg, MB, Canada; ^4^Department of Audiology, University of British Columbia, Vancouver, BC, Canada; ^5^Department of Psychological Science, University of Alberta, Edmonton, AB, Canada; ^6^Department of Human Development and Women's Studies, California State University East Bay, East Bay, CA, United States

**Keywords:** child-directed speech, infant-directed speech, positive affect, universality, cross-language perception

## Abstract

Child-directed speech, as a specialized form of speech directed toward young children, has been found across numerous languages around the world and has been suggested as a universal feature of human experience. However, variation in its implementation and the extent to which it is culturally supported has called its universality into question. Child-directed speech has also been posited to be associated with expression of positive affect or “happy talk.” Here, we examined Canadian English-speaking adults' ability to discriminate child-directed from adult-directed speech samples from two dissimilar language/cultural communities; an urban Farsi-speaking population, and a rural, horticulturalist Tseltal Mayan speaking community. We also examined the relationship between participants' addressee classification and ratings of positive affect. Naive raters could successfully classify CDS in Farsi, but only trained raters were successful with the Tseltal Mayan sample. Associations with some affective ratings were found for the Farsi samples, but not reliably for happy speech. These findings point to a complex relationship between perception of affect and CDS, and context-specific effects on the ability to classify CDS across languages.

## Introduction

Many decades of research have supported the idea that adults speak in a specialized register, with particular acoustic and linguistic features, when speaking to infants and young children than when speaking to adults (Soderstrom, 2007; Golinkoff et al., 2015). This special form of speech, known as child-directed speech (CDS), encompasses a wide variety of particular characteristics, including higher and more variable pitch, simplified vocabulary, shortened utterances, and changes to articulatory/phonetic properties, and it has been found to support early language development. Indeed, studies suggest that exposure to more and higher quality CDS is associated with faster vocabulary development, while overheard speech generally is not (Weisleder and Fernald, 2013; Ramírez-Esparza et al., 2014). However, recently a longstanding debate about the role of cultural specification in CDS has again bubbled to the surface (e.g., Ferguson, 1978; Ochs, 1982; Pye, 1986; and more recently, Golinkoff et al., 2019; Sperry et al., 2019)–i.e. to what extent is CDS (and its role in language development) universal? Although this debate is complex and multifaceted, one important component is the finding that in cultures in which CDS is not prominent, children appear to meet language development milestones on a roughly similar timeframe (Brown, 2011, 2014; Casillas et al., 2020a,b).

There are two components to the question of CDS across languages and cultures. One is that of quantity–to what extent do different cultures use the CDS register (or talk to their infants in any register)^1^ at similar rates? Although theoretically important, in the current study we set this question aside and ask a different question: How universal are the features of CDS? In other words, when adults do talk to their infants/young children (henceforth simply “infants”), however rare or common this may be, is their speech recognizable as CDS by speakers of another language?

A distinctive CDS register has been documented in a wide variety of languages, ranging from a multitude of Western and Asian languages (e.g., Fernald et al., 1989; Fernald and Morikawa, 1993; Soderstrom, 2007 and references therein), and geographically diverse places such as the Middle East (e.g., Zeidner, 1983; Farran et al., 2016) Kenya and Fiji (Broesch and Bryant, 2015) and Vanuatu (Broesch and Bryant, 2018), leading some to suggest that this register is indeed a universal feature of human interaction between caregivers and infants. More questionable, perhaps, is the idea that CDS has similar interactive functions and takes similar forms cross-culturally (e.g., Ferguson, 1978; Fernald et al., 1989). While documented similarities exist across a broad spectrum of languages and cultures, cross-linguistic and cross-cultural work suggests that there are few—if any— truly universal features of language directed to children. The lack of universals is due in large part to variation in caregivers' views about how children should be socialized as recipients and producers of language (e.g., Stross, 1972; Heath, 1983; Bernstein Ratner and Pye, 1984; Ochs and Schieffelin, 1984, 2011; Pye, 1986; Schieffelin and Ochs, 1986; Rogoff et al., 1993; Ingram, 1995; Gaskins, 2006) but has also been linked to typological variation across languages (e.g., an absence of consonant cluster simplification because the language has few clusters to begin with) or use of some CDS-related cues for other social means (e.g., high pitch use when talking to high-status adult speakers; Pye, 1986).

Across a range of unrelated cultural communities, the idea of special “babytalk” words, linguistic simplifications of any kind, or adult interpretations of infant communicative intent is seen as detrimental to children's language development or even inappropriate given children's lower social status or lack of potential as an addressee (e.g., Heath, 1983; Ochs and Schieffelin, 1984; Pye, 1986; LeVine et al., 1994; see Gaskins, 2006 for a review). With infants and toddlers alike, patterns of caregiver responsiveness to children's bids for attention also varies given cultural norms. For example, caregivers in some contexts more consistently respond to negative than positive infant vocalizations and do so more often through non-verbal than verbal means (e.g., Yucatecy Maya and Gusii vs. Euro-American caregivers; Gaskins, 1990; LeVine et al., 1994), meanwhile older children's verbalized needs are met in some contexts with responses that do not invite further contributions from the child (e.g., Quiché Maya, Kaluli, and Tseltal Maya caregivers; Stross, 1972; Ochs and Schieffelin, 1984; Pye, 1986; Brown, 2011, 2014) or are implemented via a caregiver of more proximal social status to the child (e.g., Samoa; Ochs and Schieffelin, 1984). A common thread through most of these non-urban, traditional contexts, is that the child is encouraged to meet the demands of their interactional milieu and not the other way around; in her review, Gaskins (2006) lays out at least three dimensions of child socialization that may affect CDS content and format, including caregiver ideas about: (a) the acceptability of infants broadcasting their positive/negative inner experiences, (b) in what circumstances infants are allowed to influence the actions of others, and (c) how infants can and should go about exploring the physical world. Cross-cultural variation along these and other dimensions renders affective or communicatively functional universals of CDS highly unlikely. Indeed, even similar apparent patterns of behavior may derive from different cultural motives; e.g., the lack of simplification in speech to children is done by Kaluli caregivers to support robust language development and by Samoan caregivers to maintain the status relations that permeate all other aspects of daily life (Ochs and Schieffelin, 1984).

Even among the language communities where CDS is reported to be distinct from ADS in ways that partly overlap with the distinction in English and other urban, Western linguistic contexts, there is variation in the strength and character of its implementation (e.g., Bernstein Ratner and Pye, 1984; Kitamura et al., 2001; Broesch and Bryant, 2018). Indeed, it has been suggested that North American English is a particularly extreme example of the phenomenon (Fernald et al., 1989; Shute and Wheldall, 1989), which may introduce bias in our understanding, since the majority of studies in child language come from North America. This variation calls into question how robustly universal CDS may be.

One characteristic of CDS that is highly relevant to the question of its universality is its reported relationship to positive affect. The primary prosodic characteristics of CDS, i.e., higher and more variable pitch, are also associated with the communication of positive affect or friendliness (e.g., Fernald, 1992; Kalashnikova et al., 2017), and some studies have even suggested that the well-established infant preference for listening to CDS (The ManyBabies Consortium, 2020) may primarily stem from a preference for positive affect (Singh et al., 2002). For this reason, questions about the universality of the expression of affect and the universality of CDS may be intertwined. While there is evidence that the vocal expression of emotion may be recognized above chance performance across disparate cultures (e.g., Sauter et al., 2010; Chronaki et al., 2018), there also appears to be substantial variation in its expression and perception, with advantages in perception of native-language expression (Chronaki el al.). The extent to which affect may have universal components remains controversial (see e.g., Gendron et al., 2015; Sauter et al., 2015).

In the domain of CDS, recent work by Bryant and colleagues provides evidence in favor of universality by showing that adults can identify both CDS and speaker communicative intentions across very different cultures and languages. In one study (Bryant and Barrett, 2007), adults from a Shuar (hunter-horticulturalist) village in Ecuador were able to discriminate, at about 70% accuracy, CDS from ADS samples spoken by English adults. They were also able to discriminate four categories of communicative intention (attention, prohibition, approval, comfort), with somewhat better performance in the CDS than the ADS samples. This latter result is consistent with similar findings within an English-to-English study (Fernald, 1989). Similar results to the Shuar findings were found in a later study involving adults from a Turkana (pastoralist) community in Northwestern Kenya, although there was less evidence for a role of CDS in facilitating the recognition of intention (Bryant et al., 2012).

In the current study, we add to this small body of research on cross-cultural perception of CDS. We build on the existing studies by Bryant and colleagues in several ways. First, we extend the analysis to two new languages/cultural contexts in order to add depth to the question of universality and cross-cultural similarities and differences: one an Iranian urban Farsi-speaking community (Experiment 1), and the other a small-scale subsistence Tseltal Mayan community in Southern Mexico (Experiment 2). In our study, we also “turn the tables” by asking English-speaking participants to discriminate samples from these other languages. Additionally, we add an element of ecological validity to the analysis by using audio samples that were recorded in a semi-naturalistic elicitation task (Farsi sample) and fully naturalistic realworld recordings (Mayan sample). In Experiment 2, we present data collected from trained research assistants (Experiment 2a) and from naive listeners (Experiment 2b). Finally, we explicitly examine the relationship between identification of CDS and perception of positive affective emotions. Specifically, we ask the following questions:

1. Can English speakers accurately discriminate CDS from ADS in two unfamiliar languages, Tseltal and Farsi?
2. Are speakers more likely to label speech as CDS if they perceive it to contain high positive affect (regardless of its actual status of CDS/ADS)?

In addressing these questions, we note that the questions of a potential relationship between CDS and affectively positive speech, the potential universality of CDS, and the potential universality of affective features are not straightforward to disentangle experimentally. See Table 1 for a summary of predicted outcomes given various hypotheses. In the current study, since we have no access to a “ground truth” identification of the speakers' intended affective communication, we start from the assumption (possibly unwarranted, but at least partially supported by the above-cited literature) that some degree of universal characteristics of affect are perceivable across languages–in other words that English speakers will hear happy speech produced by Farsi and Tseltal Mayan speakers as happy. We will return to this assumption in the Discussion section in order to more fully address the implications of this limitation.

**Table 1:** Overview of predicted outcomes for the present analysis, given various (simplified) ground truth conditions.

**Ground truth**		**Predictions**		
**CDS is universally tied to positive affect**	**CDS has universal prosodic feature(s)**	**Non-native labeling accuracy**	**CDS label relates to affect rating**	**Implications for CDS universality**
True	True	**High**	**High**	CDS is universally identifiable via both prosody and affect
False	False	**Low**	**High** ^***a***^	CDS is *not* universally identifiable on the basis of prosody or affect
True	False	**High**	**High**	CDS is universally identifiable via affect but not prosody
False	True	**High**	**Low**	CDS is universally identifiable via prosody but not affect

a *Here we assume that our English-speaking participants, in the absence of other options, will rely on affect in their judgments*.

## Experiment 1: Farsi

### Materials and Methods

#### Adult Participant Raters

English-speaking participant raters were recruited through the Introductory Psychology subject pool at the University of Manitoba in Winnipeg, Canada, and received course credit for their participation. The research was approved through the Psychology-Sociology Research Ethics Board of the University of Manitoba, and informed consent was obtained from each participant. Inclusion criteria included English as a primary language spoken and normal hearing. A total of 41 participants were included in the final sample. One participant was excluded after partially completing the study as they self-identified as being familiar with Farsi.

#### Stimuli

Farsi recordings were collected from a sample of mother-infant dyads in Tehran, Iran during a playgroup for mothers and babies. The research was approved through the Psychology-Sociology Research Ethics Board of the University of Manitoba, and informed consent was obtained in Farsi from each participant from an Iranian native (the 6th author) who collected the samples. Dyads were recorded in an adjacent room to the playgroup, however there is some ambient noise from the playgroup in the recordings. In total, recordings from *N* = 9 mothers were used in the rating study. Infants were aged 2–9 months.

CDS and ADS samples were collected using a semi-naturalistic task developed for the elicitation of CDS samples (The ManyBabies Consortium, 2020). For the CDS samples, mothers took a series of ten objects out of a bag and talked about the objects with their infant one at a time. The same procedure was implemented for the ADS using the Farsi-speaking researcher as the interlocutor. Recordings were later segmented and transcribed in ELAN (Wittenburg et al., 2006) by a different native Farsi speaker at the University of Manitoba. Each utterance was tagged as child-directed or adult-directed.

#### Rating Procedure

This experiment was run in the context of an honors thesis on the part of the 4th author examining the relationship between affective/emotion labels and CDS. Audio clips were segmented from the recordings using custom processing software written by our lab based on the transcripts. Utterances that had been tagged as being produced by an adult were pulled from the larger recording and turned into short wav files. Each clip was identified as to whether it was directed to an adult or the infant based on the annotator tags. These wav files were then randomly shuffled so that the clips were no longer in chronological or recording order. The recordings were then split into four relatively even stimulus groups with between 150 and 174 clips in each group.

Each participant was assigned randomly to one of the four stimulus groups. The study took 1–1.5 h to complete, and participants were offered a 5-min break halfway through to avoid effects of fatigue. Using a custom Python script developed in our lab, the utterances were presented as individual audio clips to each participant, who was asked to identify them as child-directed or adult-directed using the appropriate button, and their confidence in this rating on a scale from 0 to 4. For each clip, they were also asked to rate the extent to which the speaker in each clip exhibited the following characteristics: Happy, angry, sad, soothing, loving, exaggerated. The scale ranged from 1 to 5 with 3 as “neutral.” They were also asked to rate the extent to which the background noise was distracting (on the same scale), as a check on the quality of the audio. Background noise ratings were low indicating this was not a significant concern. An exit survey assessed their knowledge of infant-directed speech and the characteristics they used in deciding how to classify each clip (these data were not analyzed further for this study).

### Analysis

The two primary hypotheses were pre-registered on OSF prior to conducting the analyses: https://osf.io/wq5af However, because the Farsi data originate from an honors thesis project, some of the authors were aware of the findings of a similar analysis conducted on these data prior to the pre-registration.

#### Confirmatory Analyses

All statistical analyses were conducted in R with the lme4 packages (Bates et al., 2015; R Core Team, 2020) and all figures were generated with ggplot2 (Wickham, 2016).

Analysis scripts and raw anonymized data are available at https://github.com/BLLManitoba/LabellingPaperData2020. Our dependent measures Accuracy (main model) and Addressee (exploratory model) are both binary measures–correct/incorrect and cds/ads, respectively.

We used a binomial mixed-effects logistic regression of accuracy to both determine whether there are differences in English speakers' ability to identify ADS and CDS in an unfamiliar language (hypothesis 1) and whether positive affect plays a role in determining intended addressee (hypothesis 2).

The simple effects included in the main model were Addressee (cds/ads), Confidence, sounds Happy, sounds Loving, sounds Soothing, and sounds Exaggerated^2^. We also included the interaction terms between the positive affect measures and Addressee. Note that these models only take one reference level as the default for comparison for each factorial predictor (e.g., it will compare one level of the affect measure to each of the others but doesn't do full pairwise comparisons between the different levels of affect). We set the neutral rating as our reference group (affect measures). Therefore our models give us pairwise difference information between neutral rating and each of the other affect rating levels, but not for the pairwise differences between other levels (e.g., somewhat happy vs. extremely happy).

#### Exploratory Analysis

To further explore the role positive affect plays in identifying CDS compared to ADS, we fit a binomial mixed-effects logistic regression with Addressee (cds = 0, ads = 1) as our dependent measure. The simple effects in this model were positive affect measures of sounded happy, loving, soothing, and exaggerated. We again used the “*Neutral*” rating as our reference group.

### Results

**Figure 1:**
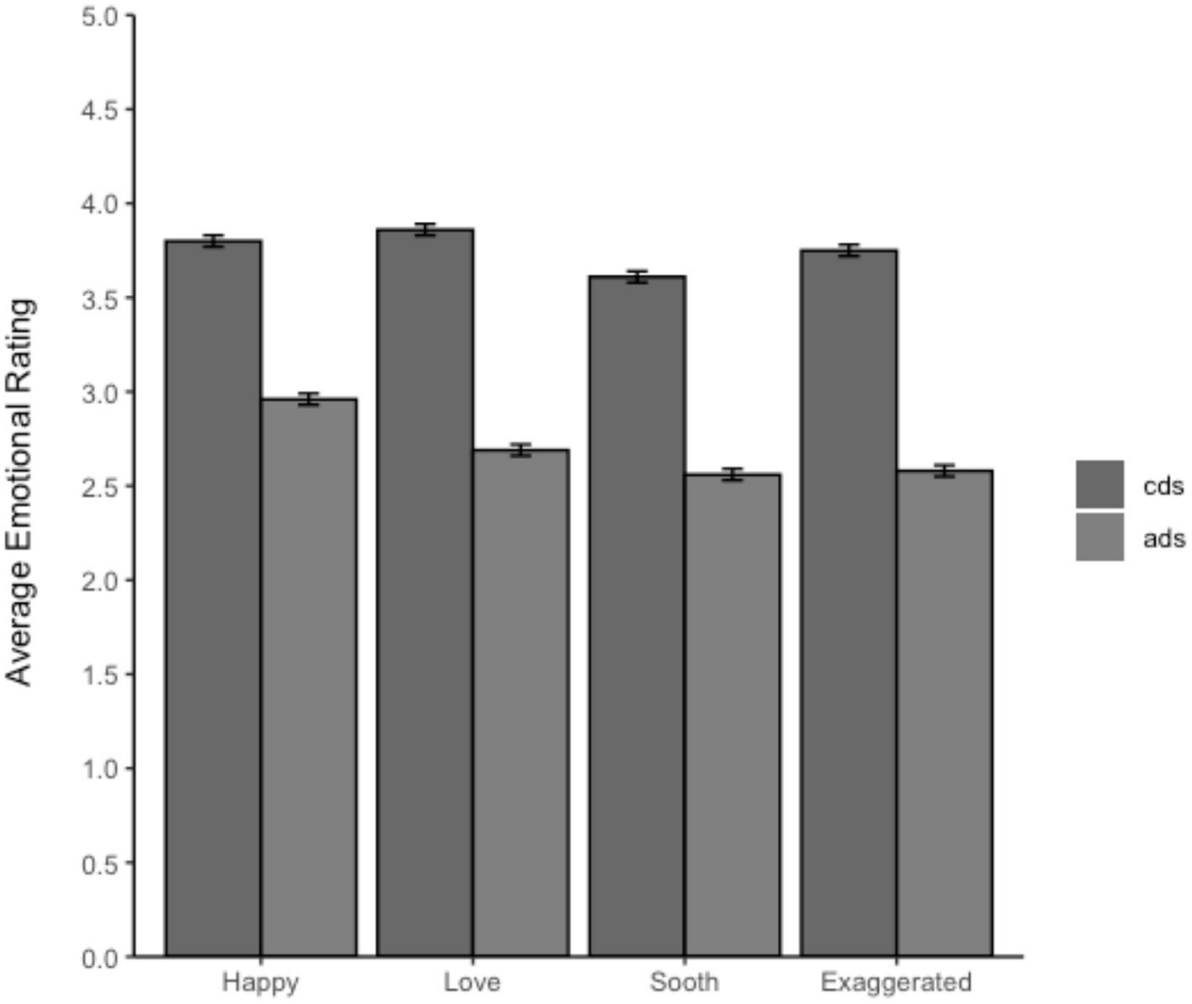
Average rating of each of the positive affect measures by addressee. The error bars represent the 95% CIs. The rating scale was 1–5 with 1 being “*extremely not sounding*” like that emotion, 3 being neutral, and 5 being “*sounded extremely*” like that emotion. Thus, average ratings >3 suggest a tendency to rate the speech as containing this emotion and values <3 suggest the speech was rated as not sounding like that emotion.

Descriptive statistics for Accuracy are shown in Table 2. Figure 1 provides a breakdown of the ratings for each affect measure by addressee. Note that the affective rating for all four measures was higher for child-directed than adult-directed speech.

**Table 2:** The Experiment 1 counts, means, and standard deviations for Accuracy broken out by cds, ads, and overall performance.

	**n**	**mean**	**sd**
**Experiment 1**			
cds	2,323	0.84	0.37
ads	3,827	0.79	0.40
overall	6,150	0.82	0.38
**Experiment 2a**			
cds	2,938	0.78	0.41
ads	2,351	0.84	0.37
overall	5,289	0.81	0.39
**Experiment 2b**			
cds	2,039	0.51	0.50
ads	592	0.52	0.50
overall	2,631	0.52	0.50

#### Confirmatory Analyses

As predicted, across all the clips participants' average accuracy identifying a speaker's intended addressee in an unfamiliar language was well above chance (82.1%, sd = 0.38). Our model of Accuracy included Addressee, Confidence, sounded Happy, sounded Sad, sounded Loving, sounded Soothing, sounded Exaggerated, the interaction terms for addressee with each of the 4 positive affect measures, and random intercepts by recording [*N* = 6150, log-likelihood = −2,179.1, overdispersion estimate = 2.61; formula: Accuracy ~ Addressee + Confidence + happy + love + sooth + exaggerate + Addressee^*^happy + Addressee^*^love + Addressee^*^sooth + Addressee^*^exaggerate + (recording)].

The participants' Accuracy significantly differed by Addressee, Confidence, and the interactions between Addressee and sounded Soothing, sounded Loving and sounded Exaggerated. Accuracy was significantly lower for ads compared to cds (*B* = −0.57, SE = 0.15, *z* = −3.81, *p* < 0.001). Participants' accuracy significantly improved with higher ratings of confidence (*B* = 0.29, SE = 0.04, *z* = 7.06, *p* < 0.001). The model also revealed significant interactions between addressee and speech that sounded soothing, loving, or exaggerated.

To fully interpret the interactions, we used planned comparisons with a Bonferonni correction (alpha adjusted to < 0.01) to determine how the specific affect ratings impacted accuracy for cds relative to ads. Across the three significant affect measures a similar pattern of results emerged. We found a significant decrease in accuracy for cds tagged as “*Somewhat not loving*” compared to ads (*B* = −0.87, SE = 0.27, *z* = −3.21, *p* < 0.01), a significant relative increase in the accuracy of cds tagged as “*Somewhat Loving*” (*B* = 1.41, *SE* = 0.23, *z* = 5.98, *p* < 0.001) and “*Extremely Loving*” (*B* = 2.07, *SE* = 0.64, *z* = 3.22, *p* < 0.01), and no differences in cds and ads that was rated as neutral or Extremely Not Loving (*p* > 0.24). For Soothing, we found a significant relative increase in the accuracy of cds tagged as “*Somewhat Soothing*” (*B* = 1.56, *SE* = 0.24, *z* = 6.57, *p* < 0.001) and “*Extremely Soothing*” (*B* = 2.99, *SE* = 0.71, *z* = 4.19, *p* < 0.001), and a marginal difference in cds and ads that was rated as neutral (*B* = −0.51, *SE* = 0.29, *z* = −1.74, *p* = 0.08). Finally, we found that accuracy significantly increased relative to ads for cds clips labeled “*Somewhat Exaggerated*” (*B* = 0.98, *SE* = 0.21, *z* = 4.78, *p* < 0.001) and “*Extremely Exaggerated*” (*B* = 2.48, *SE* = 0.57, *z* = 4.38, *p* < 0.001), and significantly decreased in accuracy for cds clips labeled “*Somewhat Not Exaggerated*” (*B* = −1.68, *SE* = 0.22, *z* = −7.71, *p* < 0.001) and “*Extremely Not Exaggerated*” (*B* = −2.24, *SE* = 0.43, *z* = −5.23, *p* < 0.001). The full results of the best-fit model are reported in Supplementary Table 1. Overall, for each of loving, soothing and exaggerated (but not happy), in general higher ratings led to higher likelihood of accurately identifying CDS compared with ADS. In other words, being rated as loving, soothing and exaggerated increased the likelihood of being labeled as CDS.

#### Exploratory Analysis

To probe the effect of emotion rating on cds labeling more directly, our exploratory model used addressee (as identified by the raters, not ground truth) as the dependent measure and included the predictors sounded happy, sounded loving, sounded soothing, sounded exaggerated and the random intercepts by recording [*N* = 6,150, log-likelihood = −2,660.8, overdispersion estimate = 23.05; formula = Addressee ~ happy + love + sooth + exaggerate + (recording)].

**Figure 2:**
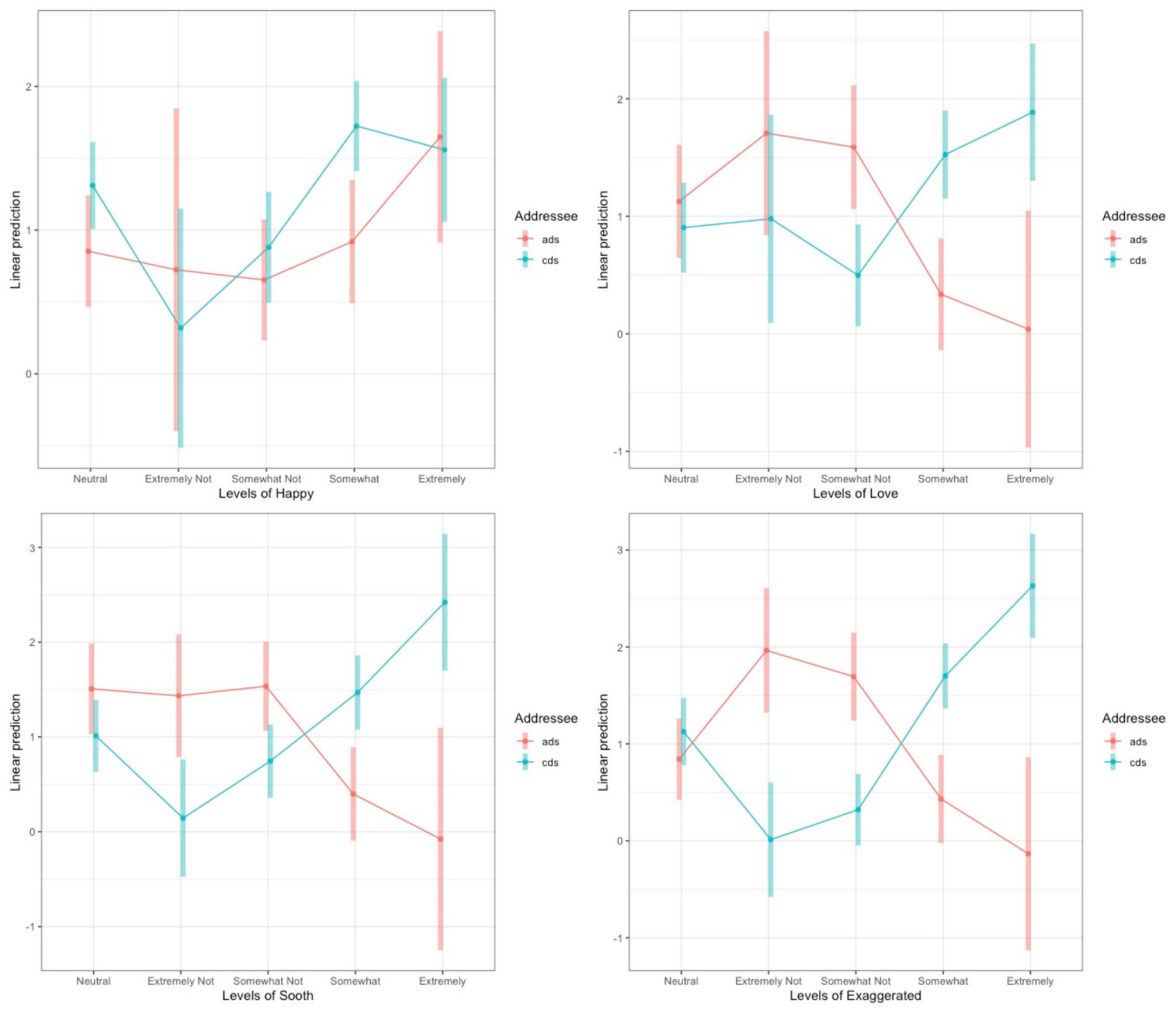
Levels of sounded Happy (top left), sounded Loving (top right), sounds Soothing (bottom left), and sounded Exaggerated (bottom right) for the interactions with Addressee for Experiment 1. The log odds of each affect level are plotted on the y-axis and the shaded areas represent the 95% CIs. The interactions between Addressee and Loving, Soothing and Exaggerated were significant. Neutral is graphed on the left as it was the reference level.

The model revealed that speech tagged as Extremely Not Happy was marginally more likely to be identified as cds compared to ads (*B* = −0.52, *SE* = 0.31, *z* = −1.66, *p* = 0.098), speech tagged as “*somewhat not happy”* was significantly more likely to be ads (*B* = 0.55, *SE* = 0.10, *z* = 5.49, *p* < 0.001), clips labeled “*Somewhat Happy*” were significantly more often labeled cds (*B* = −0.69, *SE* = 0.09, *z* = −8.07, *p* < 0.001) and “*Extremely Happ*y” clips were marginally more often cds (*B* = −0.34, *SE* = 0.18, *z* = −1.91, *p* = 0.056). We found that cds clips were significantly more frequently labeled “*Somewhat Loving*” (*B* = −0.71, *SE* = 0.10, *z* = −6.86, *p* < 0.001) and “*Extremely Loving*” (*B* = −1.68, *SE* = 0.28, *z* = −6.10, *p* < 0.001). For Soothing, we found that cds clips were significantly more frequently labeled “*Somewhat soothing*” (*B* = −0.58, *SE* = 0.11, *z* = −5.55, *p* < 0.001) and “*Extremely soothing*” (*B* = −1.51, *SE* = 0.29, *z* = −5.24, *p* < 0.001), and clips labeled “*Somewhat Not Soothing*” were significantly more frequently ads (*B* = 0.25, *SE* = 0.11, *z* = 2.28, *p* < 0.05). Finally, we found that cds clips were significantly more frequently labeled “*Somewhat Exaggerated*” (*B* = −1.14, *SE* = 0.09, *z* = −12.53, *p* < 0.001) and “*Extremely Exaggerated*” (*B* = −2.54, *SE* = 0.24, *z* = −10.68, *p* < 0.001), and significantly more frequently ads was labeled “*Somewhat Not Exaggerated*” (*B* = 0.39, *SE* = 0.09, *z* = 4.40, *p* < 0.001) and “*Extremely Not Exaggerated*” (*B* = 0.94, *SE* = 0.17, *z* = 4.88, *p* <0.001). Figure 2 shows the interaction between the four affect measures and Addressee and the full results of the best-fit model are reported in Supplementary Table 2. Overall, these results are consistent with the confirmatory analysis that being rated as loving, soothing and exaggerated increased the likelihood of being labeled as CDS, while the results for happy were more mixed.

## Experiment 2: Tseltal Mayan

Experiment 1 provided evidence in favor of our first hypothesis, that English-speaking adults could discriminate CDS from ADS in an unfamiliar language (Farsi). It also showed that there was a relationship between those judgments and raters' perception that the clips were “loving,” “soothing” and/or “exaggerated” (but less clearly for “happy”).

In Experiment 2, we conducted a parallel analysis on speech from a Tseltal Mayan sample. These clips differed from the Farsi clips on a number of characteristics in addition to the language: They were highly naturalistic samples from everyday life (vs. semi-naturalistic speech from an elicitation task), and came from a rural, horticulturalist community in Mexico vs. a urban industrialized community in Iran. In Experiment 2, for the CDS/ADS distinction, we also present rating data collected from a small number of trained research assistants (Experiment 2a) in addition to the naive participant raters (Experiment 2b).

### Materials and Methods

#### Adult Participant Raters

For Experiment 2a, raters were five trained research assistant transcriber-annotators in the Baby Language Lab at the University of Manitoba. All spoke Canadian English as their primary language and had normal hearing. The ratings were collected as part of their normal duties processing the audio files as transcriber-annotators.

For Experiment 2b, participant raters were recruited as in Experiment 1. Inclusion criteria included English as a primary language spoken and normal hearing. Thirty-two participants rated the samples for CDS/ADS and a separate 32 participants rated the samples for affect. Two additional participants' data were excluded: One started the experiment but decided to stop labeling after a handful of clips. Another participant in the affect group completed a unique set of clips designed to fill in missing data that the other participants in the affect group had not labeled during the experiment. However, it was ultimately decided not to include these data as it would have added problematic complexity to the model.

#### Stimuli

The Tseltal recordings were collected in 2015 from children growing up in a rural, horticulturalist Tseltal Mayan village in the Chiapas highlands of Southern Mexico. The research was approved through the Radboud University Social Sciences Ethics Committee and informed consent was collected verbally and interactively in Tseltal from the members present in each recorded household. Children and their families were visited on the morning of the recording day by the 2nd author and a local research assistant who would conduct informed consent and give instructions before fitting the target child with an elastic vest containing a lightweight stereo audio recorder (Olympus WS-832) across the chest and a miniature photo camera (Narrative Clip 1) on the shoulder strap. The researchers then left the child and family to go about their ordinary business for the day, returning 7–11 h later to collect the recording equipment. The original corpus contains recordings from 55 children under 4;0. A subset of *N* = 10 children under 3;0 were selected for manual annotation of language activity (see Brown, 2011, 2014 and Casillas et al., 2020b for more information regarding the Tseltal cultural context). These recordings are available via the Casillas HomeBank repository (Casillas et al., 2017).

Nine 5-min clips were randomly selected from throughout each of the 10 children's recording days and were then fully annotated for all hearable speech from the target child and others in their environment by the 2nd author and a local research assistant. Each stretch of non-target-child speech was assigned to a speaker identity (e.g., the child's aunt/brother/etc.), annotated for addressee (e.g., target-child-directed, other-child-directed, adult-directed, etc.), transcribed, and loosely translated into Spanish (Casillas et al., 2020b) in ELAN (Wittenburg et al., 2006). In the present study we used the speaker identity and intended addressee annotations to extract relevant clips to present to English-speaking participants (see below).

#### Rating Procedure: Trained Raters

These data were collected as part of the general processing and classification of the Tseltal Mayan samples described above in preparation for future studies (such as Experiment 2b). Stimuli were generated by running custom software similar to the software used to process the Farsi language recordings. The software took the previously labeled and tagged longform Tseltal language recordings and generated short wav files of utterances directed at adults and children that were made by male and female adult speakers. In the current analysis only the data from female speakers was included for greater consistency with the analyses in Experiments 1 and 2b. In total, there were 5,291 clips that met these criteria. However, 2 clips were missed during the analysis, so a total of 5,289 clips were included in the sample. The order of the clips was not randomized. Trained research assistants labeled the clips according to their perception of whether the utterances were adult or child directed, or whether the clip was too noisy and should be classified as junk (*N* = 210; due to the naturalistic nature of these recordings in multi-speaker, and variable indoor and outdoor rural environments, there was a high degree of ambient noise). These latter were excluded from further analysis. The research assistant also gave a rating of their confidence on a scale of one to five, with one being not at all confident, and five being fully confident in their assessment of the clip's directive target. Affective ratings were not collected in this analysis.

#### Rating Procedure: Naive Raters

This experiment was run as part of an undergraduate research project examining the relationship between maternal and infant vocal affect in naturalistic interactions. A total of 2,631 clips were randomly sub-selected from those in 2a (excluding noisy clips). Clips were generated from solely female adult speakers' utterances that were tagged as being directed at an adult or a child. We then randomly grouped the clips into through 3 roughly equal groups. Participants were randomly assigned to one of the sets. Half of the participants were instructed to label addressee (ads/cds group) and the other half were assigned to label the speakers' affect (affect group) to ultimately form 32 dyads. Infant vocal affect was also rated by a separate group of participants but those data are not reported here.

The study took 45–60 min to complete. Each participant would rate clips until they were finished or their hour time slot was up. Using a custom Python script based on that of Experiment 1, the utterances were presented as individual audio clips to each participant. Participants in the ADS/CDS group were asked to identify them as child-directed or adult-directed using the appropriate button, and their confidence in this rating on a scale from 0 to 4. Participants in the affect group rated the clips according to the categories of loving, soothing, happy, and excited, with a scale from 1 “neutral” to 5 “extremely [category].” Note that these categories are similar, but not identical, to those used in Experiment 1.

### Analysis

As noted above, the two primary hypotheses were pre-registered on OSF prior to conducting the analyses: https://osf.io/wq5af.

#### Confirmatory Analyses (Trained Raters)

Similar to Experiment 1, we used a binomial mixed-effects logistic regression of accuracy to determine whether there are differences in English speakers' ability to identify ADS and CDS in an unfamiliar language (hypothesis 1). The simple effects included in the main model were Addressee (cds/ads) and Confidence (from the CDS/ADS group).

#### Confirmatory Analyses (Naive Raters)

Confirmatory analyses were conducted parallel to those described for the Farsi data. The simple effects included in the main model were Addressee (cds/ads), Confidence, sounds Happy, sounds Loving, sounds Soothing, and sounds Excited and the interaction terms of each affective measure with Addressee. We again set the neutral rating as our reference group (affect measures), though note that neutral in this model was the lowest rating (1) rather than the middle rating (3). As a reminder, the data from Addressee and from affect ratings were collected from different (paired) participants in this model.

#### Exploratory Analysis (Naive Raters)

As with Experiment 1, we fit a binomial mixed-effects logistic regression with Addressee (cds = 0, ads = 1) as our dependent measure. The simple effects in this model were positive affect measures of sounded happy, loving, soothing, and excited. We again used the “*Neutral*” rating as our reference group.

### Results

**Figure 3:**
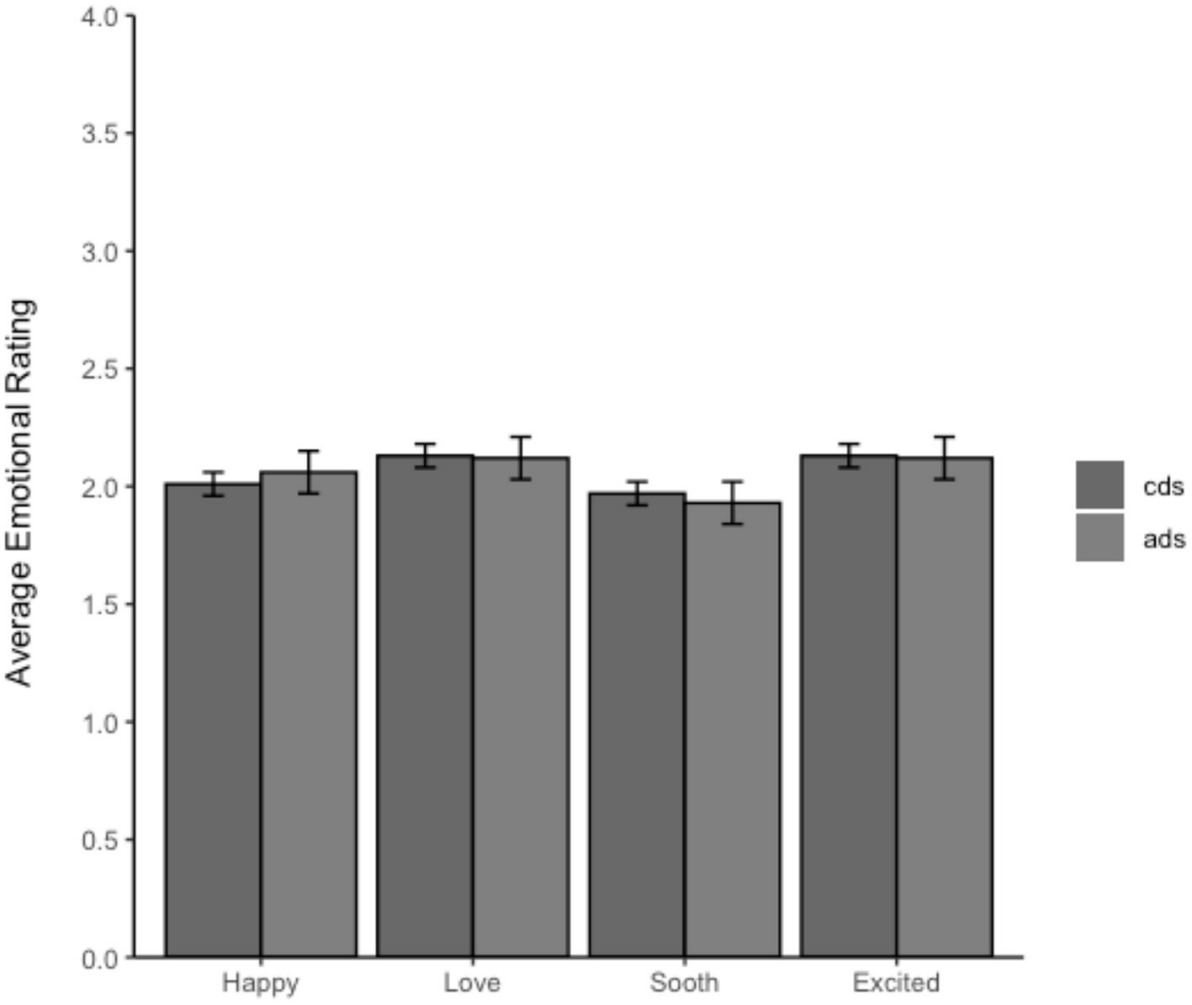
Average ratings of the positive affect measures by addressee from Experiment 2b. The error bars represent the 95% CIs. The ratings on this scale were from 1 to 5 with 1 being neutral and 5 being “*sounded extremely*” like that emotion.

Descriptive statistics for Accuracy are shown in Table 2, Figure 3 provides a breakdown of the ratings in Experiment 2b for each affect measure by addressee. Note that the affective rating for all four measures did not differ between child-directed and adult-directed speech.

#### Confirmatory Analyses (Trained Raters)

Similar to Experiment 1, across all the clips raters' average accuracy identifying a speaker's intended addressee in an unfamiliar language was well above chance at 80.1% (sd = 0.39, see Figure). However, unlike the participants in Experiment 1 the labellers in Experiment 2 had higher accuracy tagging ads clips (*M* = 0.84, SD = 0.37) compared to cds clips (*M* = 0.78, SD = 0.41). Our binomial mixed-effects logistic regression of Accuracy included Addressee and Confidence as fixed, and random intercepts by recording [*N* = 5,289, log-likelihood = −2,332.4, overdispersion estimate = 1.32; formula = Accuracy ~ Addressee + Confidence + (recording)]. Accuracy was significantly lower for cds compared to ads (*B* = −0.83, SE = 0.09, z = −9.44, *p* < 0.001). Higher confidence predicted improved accuracy (*B* = 0.89, SE = 0.05, *z* = 17.73, *p* < 0.001).

#### Confirmatory Analyses (Naive Raters)

Counter to our prediction and unlike the participants in the prior two analyses, the naive labellers' average accuracy identifying a speaker's intended addressee in an unfamiliar language was approximately at chance (*M* = 0.52, SD = 0.50) and this was true for both cds (*M* = 0.51, SD = 0.50) and ads (*M* = 0.52, SD = 0.50). To explore the participants' performance statistically, we used a similar model structure from Experiment 1. Our model of Accuracy included fixed effects for Addressee, Confidence, sounded Happy, sounded Loving, sounded Soothing, sounded Excited, the interaction terms for addressee and the 4 positive affect measures, and random intercepts by recording [*N* = 2,631, log-likelihood = −1757.3, overdispersion estimate = 2.87; formula = Accuracy ~ Addressee + Confidence + happy + love + sooth + excited + Addressee^*^happy + Addressee^*^love + Addressee^*^sooth + Addressee^*^excited + (recording)].

The participants' Accuracy significantly differed by Addressee, Confidence, and the interaction between Addressee and sounded Excited. Accuracy was significantly lower for cds compared to ads (*B* = −0.79, SE = 0.21, *z* = −3.83, *p* < 0.001). Surprisingly, there was a decrease in participants' accuracy the higher they rated their confidence (*B* = −0.14, SE = 0.21, *z* = −3.88, *p* < 0.001). The model also revealed a significant interaction between addressee and speech that sounded excited.

**Figure 4:**
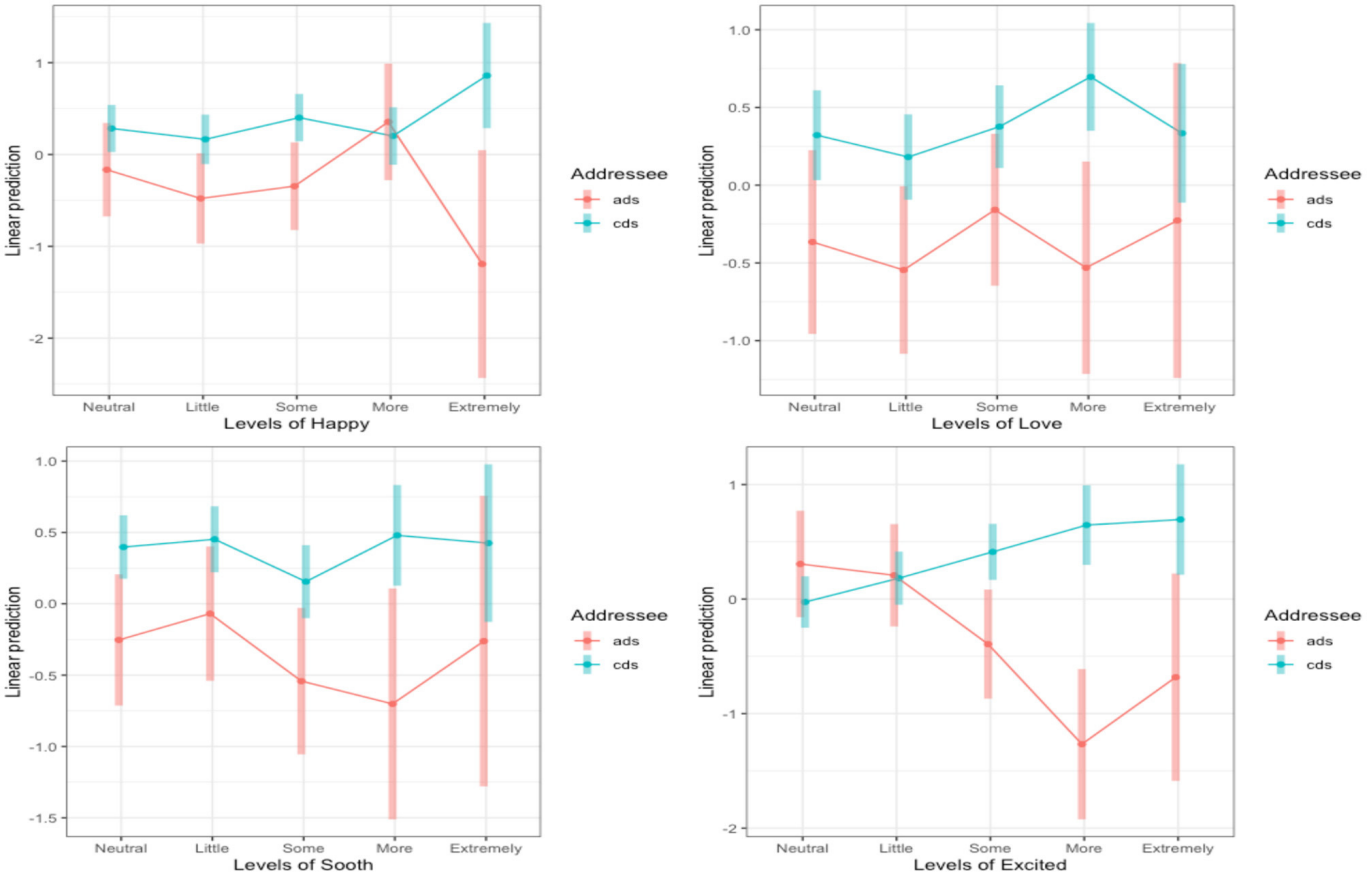
Levels of sounded Happy (top left), sounded Loving (top right), sounds Soothing (bottom left), and sounded Excited (bottom right) for the interactions with Addressee in Experiment 2b. The log odds of each affect level are plotted on the y-axis and the shaded areas represent the 95% CIs. Only the interaction between Addressee and Excited was significant.

To fully interpret the interaction, we again used planned comparisons with a Bonferonni correction (alpha adjusted to < 0.01) to determine how the specific affect ratings impacted accuracy for cds and ads. We found that accuracy significantly increased for cds clips labeled “*Somewhat Excited*” (*B* = 0.81, *SE* = 0.27, *z* = 2.96, *p* < 0.01), “*More Excited*” (*B* = 1.91, *SE* = 0.38, *z* = 5.05, *p* < 0.001), and clips labeled “*Extremely Excited*” (*B* = 1.38, *SE* = 0.52, *z* = 2.63, *p* < 0.01). There were no differences at the “*Neutral”* and “*little excited”* levels (ps > 0.21). Figure 4 shows the interaction between the four affect measures and Addressee and the full results of the best-fit model are reported in Supplementary Table 3.

#### Exploratory Analysis (Naive Raters)

As in Experiment 1, to further explore the role that positive affect may play in identifying child-directed speech compared to adult-directed speech, we fit a mixed-effects with Addressee (cds = 0, ads = 1) as our dependent measure. We included the predictors sounded happy, sounded loving, sounded soothing, sounded excited and random intercepts by recording [*N* = 2,631, log-likelihood = −1141.5, overdispersion estimate = 5.15; formula = Addressee ~ happy + love + sooth + excited + (recording)]. The model revealed that clips labeled “*Extremely soothing*” were significantly more likely to be labeled as ads compared to cds (*B* = 1.00, *SE* = 0.35, *z* = 2.84, *p* < 0.01). The full results of the best-fit model are reported in Supplementary Table 4.

## Discussion

### Summary of Findings

Across 3 experiments, we examined the ability of Canadian English-speaking adults to identify CDS in two unfamiliar languages/communities, and the relationship between their judgments and measures of emotion/positive affect. Results of the first Experiment, with a semi-naturalistic sample of speech from Farsi-speaking mothers, found high accuracy for naive raters in identifying both ADS and CDS, with somewhat higher accuracy for CDS. Identification as CDS was correlated with higher rated levels of “loving,” “soothing,” and “exaggerated” speech characteristics, but not clearly with “happy” speech. In a second analysis rating speech from a Tseltal Mayan community as ADS or CDS, trained research assistants also showed high rates of accuracy, although accuracy was higher for ADS than CDS. However, a third experiment with naive raters assessing these same Tseltal Mayan samples found very poor accuracy, and an inverse relationship between confidence and accuracy (i.e., more confident ratings were less likely to be accurate). In this last study, identification as CDS was related to higher “excited” scores, but was not reliably associated with the other characteristics.

### Implications for Universality of CDS Characteristics

If we first consider the first two experiments (i.e., Experiment 1 and 2a) on their own, our findings support the idea that CDS is identifiable across different languages/cultures (at least, these specific ones). They also provide support for a relationship between affect and CDS (at least for Farsi, as affect was not rated in Experiment 2a). These findings are largely consistent with the prior work by Bryant and colleagues (Bryant and Barrett, 2007; Bryant et al., 2012). However, before delving into the details of Experiment 2b, a number of nuances to these basic findings must be articulated.

First, based on these findings alone, we cannot differentiate between two possible interpretations of these findings (see Table 1). In one interpretation, CDS is both tied to positive affect and contains universal expressions unique to the CDS register (i.e., separate from a general expression of positive affect). In this interpretation, raters used both affective characteristics and prosodic characteristics unique to CDS in their judgments. However, it is equally possible that raters made their judgments solely on the basis of affect and not on any prosodic characteristics of CDS unrelated to affect.

Second, the findings with respect to the happiness rating are unexpected and intriguing. Recall that prior work on the relationship between affect and CDS has suggested that infants' attraction to CDS is mediated by “happy talk” (Singh et al., 2002). It is therefore of interest that “happy” talk was not reliably associated with raters' judgments of CDS, while characteristics of loving, soothing and exaggerated were. This is consistent with prior research suggesting that various affective states are communicated with CDS (Fernald, 1989), and suggests that “happy” talk *per se* may not be a driving factor in the perception of CDS.

We next turn to the findings of Experiment 2b. These findings do not fit neatly with any of the patterns predicted in Table 1 we found both poor performance in discriminating CDS from ADS, and little relationship between the affective characteristics and rating of CDS, with the exception of excited speech (and less reliably inversely with soothing speech in the exploratory analysis). Of note, level of confidence was inversely related to performance, suggesting that the raters were relying on a somewhat systematic, but inaccurate, metric for CDS. As a first pass, these findings can be interpreted as a failure to identify CDS in the Tseltal Mayan sample. Harder to ascertain is why, particularly relative to the success with naive raters on Farsi in Experiment 1 and with trained research assistants on Tseltal Mayan in Experiment 2a. One salient possible interpretation is that the cultural context of Tseltal Mayan is more distinct from Canadian English language/culture than that of Farsi, making identification of CDS more challenging. However, the success of the research assistants suggests that this identification is not impossible, just hard. Having spent time working with naturalistic language files of this type may have given the research assistants an “edge” in detecting subtle cues based on their greater experience with these kinds of samples despite their lack of access to the conversational context or meaning of the speech. It is important to note that there are other possible reasons for the apparent greater difficulty in the Tseltal Mayan samples, however. For example, the samples were taken in a fully naturalistic, everyday life, context, whereas the elicitation task of the Farsi samples may have served to exaggerate some characteristics of CDS. Second, there was an age difference between the Farsi and the Tseltal Mayan infants, which may influence the type and degree of CDS used. Moreover, the Tseltal Mayan samples included both speech to the target child and to other nearby children, whereas the Farsi samples were restricted to the infants under study, which could also have affected the nature of the speech samples.

The reason that the pattern of results for Experiment 2b did not appear in our Table 1 is because of two assumptions inherent in the predictions. First, that emotions are detectable across cultures, and second that in the absence of salient direct cues to CDS, raters would rely on their judgments of the emotional characteristics. These findings suggest that the first of these assumptions, and to an extent the second, is incorrect. Unfortunately, we do not have ground truth measures of the intended affective communication in the Tseltal Mayan samples (or from a third party Tseltal Mayan listener), so it is not possible to determine whether the raters were perceiving happiness, lovingness, soothingness or excitedness in the same way as a Tseltal Mayan speaker, nor whether such cues existed in the samples at all. Moreover, the affective judgments made in Experiment 2b were made by a separate group of participants than those making the CDS/ADS discrimination, so we cannot ask this question at the level of individual participants, but only at the level of group based judgments regarding affect and CDS. Nonetheless, our findings suggest that lovingness, soothingness and happiness were not used by the raters in making their judgment regarding whether an utterance was CDS or ADS. Instead, the raters relied at least in part on the degree of excitedness they perceived in the speech–but reliance on this characteristic did not lead them to accurate judgments.

### Limitations, Conclusions, and Future Directions

In interpreting these findings, it is important to point out that we did not conduct a systematic or fulsome exploration of how classification of CDS/ADS (and its relationship to positive affect) might vary across languages, language typologies, cultural contexts or communities. Our analyses were conducted purely over samples of convenience regarding two non-English languages to which we had access. These languages and communities differ in important ways from North American culture and language and from each other, but are far from representing the vast diversity of infant linguistic experience. Moreover, differences both in the context over which the speech was sampled and the methodological details in the ratings collection limit our ability to directly compare the findings across the two analyses and identify with certainty the reason for the differential findings. In particular, scripted speech (e.g., Singh et al., 2002) or semi-structured, often object-focused, activities such as those used in the elicitation task with the Farsi sample (e.g., Fernald, 1989; The ManyBabies Consortium, 2020) underly much of the work investigating CDS in Western contexts. However, the register is recognizably present in other data types, including brief free-play sessions (e.g., Kitamura et al., 2001) and daylong recordings similar to those used in our Tseltal Mayan sample (e.g., Bergelson et al., 2019), which cross a range of at-home naturalistic circumstances.

Nonetheless, these three analyses, together with the prior work by Bryant and colleagues, are an important first step in teasing apart these thorny questions of the universality of CDS and the relationship between CDS and the perception of affect. With the above limitations in mind, our findings suggest that the answer to these questions is not straightforward. Our findings are consistent with the decades-long literature on the acoustic and linguistic characterizations of CDS itself (e.g. Fernald et al., 1989; Soderstrom, 2007)—we see evidence both for shared properties and variation across languages in the crosslanguage perception of CDS. Gaining a window into the extent to which true “universals” may be established will require a much broader and systematic examination across different language and cultural typologies. Our findings also suggest that perception of CDS (and affect) outside of one's own language may be highly sensitive to the context in which the speech was collected. This may be particularly true due to the laboratory-based, decontextualized conditions in which our raters made their judgments. Our findings further suggest that affect does affect raters' perception of CDS, but not in a simple way–contrary to our expectations, we did not find a consistent relationship across either study between ratings of happiness and raters' perception of CDS.

One additional question left unanswered by the research so far is the specific characteristics adult raters use to make their judgments. Our starting assumption is that pitch characteristics play a primary role in these judgments, although the specific acoustic features underlying this relationship remain unidentified. Both the current study and the prior work by Bryant and colleagues, in providing evidence for cross-language identification, rule out the possibility that an understanding of the substantive content of the speech (e.g., topic) are necessary. However, it is possible that other characteristics such as articulatory/phonetic features may play a role.

In sum, elements of CDS appear discriminable across vastly different languages and cultural contexts, and this discrimination is tied to affective characteristics of the speech. However, the relationship between affective and other properties of speech and the characterization of CDS is complex and highly context-sensitive.

## Data Availability Statement

The datasets and analytic scripts used in this study can be found https://github.com/BLLManitoba/LabellingPaperData2020.

## Ethics Statement

The studies involving human participants were reviewed and approved by the Psychology-Sociology Research Ethics Board, University of Manitoba. The participants provided their written informed consent to participate in this study.

## Author Contributions

AS and MC collected and contributed the speech samples. AB and JB conducted Experiment 1. MG and SM conducted Experiment 2. JB conducted the analyses. MS, JB, and MC were primarily responsible for writing the manuscript. All authors approved the final draft.

## Conflict of Interest

The authors declare that the research was conducted in the absence of any commercial or financial relationships that could be construed as a potential conflict of interest.

## Publisher's Note

All claims expressed in this article are solely those of the authors and do not necessarily represent those of their affiliated organizations, or those of the publisher, the editors and the reviewers. Any product that may be evaluated in this article, or claim that may be made by its manufacturer, is not guaranteed or endorsed by the publisher.

## Footnotes

^1^In the literature, this term is often used interchangeably to refer to speech directed toward children regardless of linguistic or acoustic characteristics, and speech that contains the expected register features regardless of addressee.

^2^The preregistered model accidentally included one negative affect measure (sounds sad)–here we present the model without this effect for the sake of clarity, but inclusion does not change the results.

## References

[B1] Bates D. Mächler M. Bolker B. Walker S. (2015). Fitting linear mixed-effects models using lme4. J. Stat. Softw. 67, 1–48. 10.18637/jss.v067.i01

[B2] Bergelson E. Casillas M. Soderstrom M. Seidl A. Warlaumont A. S. Amatuni A. (2019). What do North American babies hear? a large-scale cross-corpus analysis. Dev. Sci. 22:e12724. 10.1111/desc.12724 30369005 PMC6294666

[B3] Bernstein Ratner N. B. Pye C. (1984). Higher pitch in BT is not universal: acoustic evidence from Quiche Mayan. J. Child Lang. 11, 515–522. 10.1017/S0305000900005924 6501462

[B4] Broesch T. Bryant G. A. (2018). Fathers' infant-directed speech in a small-scale society. Child Dev. 89, e29–e41. 10.1111/cdev.12768 28239835

[B5] Broesch T. L. Bryant G. A. (2015). Prosody in infant-directed speech is similar across western and traditional cultures. J. Cogn. Dev. 16, 31–43. 10.1080/15248372.2013.833923

[B6] Brown P. (2011). The cultural organization of attention, in Handbook of Language Socialization, eds Duranti A. Ochs E. Schieffelin B. B. (Malden, MA: Wiley-Blackwell), 29–55.

[B7] Brown P. (2014). The interactional context of language learning in Tzeltal, in Language in Interaction: Studies in Honor of Eve V. Clark, eds Arnon I. Casillas M. Kurumada C. Estigarribia B. (Amsterdam, NL: John Benjamins), 51–82.

[B8] Bryant G. A. Barrett H. C. (2007). Recognizing intentions in infant-directed speech: evidence for universals. Psychol. Sci. 18, 746–751. 10.1111/j.1467-9280.2007.01970.x 17680948

[B9] Bryant G. A. Liénard P. Clark Barrett H. (2012). Recognizing infant-directed speech across distant cultures: evidence from Africa. J. Evol. Psychol. 10, 47–59. 10.1556/JEP.10.2012.2.1

[B10] Casillas M. Brown P. Levinson S. C. (2017). Casillas HomeBank Corpus. 10.21415/T51X12

[B11] Casillas M. Brown P. Levinson S. C. (2020a). Early language experience in a Papuan community. J. Child Lang. 1–23. 32988426 10.1017/S0305000920000549

[B12] Casillas M. Brown P. Levinson S. C. (2020b). Early language experience in a Tseltal Mayan village. Child Dev. 91, 1819–1835. 10.1111/cdev.13349 31891183

[B13] Chronaki G. Wigelsworth M. Pell M. D. Kotz S. A. (2018). The development of cross-cultural recognition of vocal emotion during childhood and adolescence. Sci. Rep. 8, 1–17. 10.1038/s41598-018-26889-1 29904120 PMC6002529

[B14] Farran L. K. Lee C. C. Yoo H. Oller D. K. (2016). Cross-cultural register differences in infant-directed speech: an initial study. PLoS ONE 11:e0151518. 10.1371/journal.pone.0151518 26981626 PMC4794163

[B15] Ferguson C. A. (1978). Talking to children: a search for universals, in Universals of Human Language: Vol. 1 Method and Theory, ed Greenberg J. H. (Stanford, CA: Stanford University Press), 203–224.

[B16] Fernald A. (1989). Intonation and communicative intent in mothers' speech to infants: is the melody the message? Child Dev. 60, 1497–1510. 10.2307/1130938 2612255

[B17] Fernald A. (1992). Human vocalizations to infants as biologically relevant signals: An evolutionary perspective, in The Adapted Mind: Evolutionary Psychology and the Generation of Culture, eds Barkow J. Cosmides L. (New York, NY: Oxford University Press), 391–428.

[B18] Fernald A. Morikawa H. (1993). Common themes and cultural variations in Japanese and American mothers' speech to infants. Child Dev. 64, 637–656. 10.2307/1131208 8339686

[B19] Fernald A. Taeschner T. Dunn J. Papousek M. de Boysson-Bardies B. Fukui I. (1989). A cross-language study of prosodic modifications in mothers' and fathers' speech to preverbal infants. J. Child Lang. 16, 477–501. 10.1017/S0305000900010679 2808569

[B20] Gaskins S. (1990). Mayan exploratory play and development (Ph.D. dissertation). Department of Education (Educational Psychology), University of Chicago, Chicago, IL, United States.

[B21] Gaskins S. (2006). Cultural perspectives on infant-caregiver interaction, in Roots of Human Sociality: Culture, Cognition, and Interaction, eds Enfield N. J. Levinson S. C. (Oxford: Berg), 279–298.

[B22] Gendron M. Roberson D. Barrett L. F. (2015). Cultural variation in emotion perception is real: a response to Sauter, Eisner, Ekman, and Scott (2015). Psychol. Sci. 26, 357–359. 10.1177/0956797614566659 25608863 PMC5497728

[B23] Golinkoff R. M. Can D. D. Soderstrom M. Hirsh-Pasek K. (2015). (Baby) talk to me: the social context of infant-directed speech and its effects on early language acquisition. Curr. Dir. Psychol. Sci. 24, 339–344. 10.1177/0963721415595345

[B24] Golinkoff R. M. Hoff E. Rowe M. L. Tamis-LeMonda C. S. Hirsh-Pasek K. (2019). Language matters: denying the existence of the 30-million-word gap has serious consequences. Child Dev. 90, 985–992. 10.1111/cdev.13128 30102419 PMC10370358

[B25] Heath S. B. (1983). Learning how to talk in Trackton (Ch. 3), in Ways With Words (Cambridge: Cambridge University Press), 73–112.

[B26] Ingram D. (1995). The cultural basis of prosodic modifications to infants and children: a response to Fernald's universalist theory. J. Child Lang. 22, 223–233. 10.1017/S0305000900009715 7759581

[B27] Kalashnikova M. Carignan C. Burnham D. (2017). The origins of babytalk: smiling, teaching or social convergence? R. Soc. Open Sci. 4:170306. 10.1098/rsos.170306 28878980 PMC5579095

[B28] Kitamura C. Thanavishuth C. Burnham D. Luksaneeyanawin S. (2001). Universality and specificity in infant-directed speech: pitch modifications as a function of infant age and sex in a tonal and non-tonal language. Infant Behav. Dev. 24, 372–392. 10.1016/S0163-6383(02)00086-3

[B29] LeVine R. A. Dixon S. LeVine S. Richman A. Leiderman P. H. Keefer C. H.. (1994). Child Care and Culture: Lessons From Africa. Cambridge: Cambridge University Press.

[B30] Ochs E. (1982). Talking to children in Western Samoa. Lang. Soc. 11, 77–104. 10.1017/S0047404500009040

[B31] Ochs E. Schieffelin B. B. (1984). Language acquisition and socialization: three developmental stories and their implications, in Culture Theory: Essays on Mind, Self, and Emotion, eds Schweder R. A. LeVine R. A. (Cambridge: Cambridge University Press), 276–320.

[B32] Ochs E. Schieffelin B. B. (2011). The theory of language socialization, in The Handbook of Language Socialization, eds Duranti A. Ochs E. Schieffelin B. B. (Chichester: A John Wiley & Sons, Ltd),1–21.

[B33] Pye C. (1986). Quiché Mayan speech to children. J. Child Lang. 13, 85–100. 10.1017/S.0305000900000313 3949901

[B34] R Core Team (2020). R: A Language and Environment for Statistical Computing. Vienna: R Foundation for Statistical Computing.

[B35] Ramírez-Esparza N. García-Sierra A. Kuhl P. K. (2014). Look who's talking: speech style and social context in language input to infants are linked to concurrent and future speech development. Dev. Sci. 17, 880–891. 10.1111/desc.12172 24702819 PMC4188803

[B36] Rogoff B. Mistry J. Gönc,ü A. Mosier C. Chavajay P. Heath S. B. (1993). Guided participation in cultural activity by toddlers and caregivers. Monogr. Soc. Res. Child Dev. 58, 1–179. 10.2307/1166109 8284000

[B37] Sauter D. A. Eisner F. Ekman P. Scott S. K. (2010). Cross-cultural recognition of basic emotions through nonverbal emotional vocalizations. Proc. Nat. Acad. Sci. U.S.A. 107, 2408–2412. 10.1073/pnas.0908239106 20133790 PMC2823868

[B38] Sauter D. A. Eisner F. Ekman P. Scott S. K. (2015). Emotional vocalizations are recognized across cultures regardless of the valence of distractors. Psychol. Sci. 26, 354–356. 10.1177/0956797614560771 25608864 PMC4361354

[B39] Schieffelin B. B. Ochs E. (1986). Language socialization. Annu. Rev. Anthropol. 15, 163–191. 10.1146/annurev.an.15.100186.001115

[B40] Shute B. Wheldall K. (1989). Pitch alterations in British motherese: some preliminary acoustic data. J. Child Lang. 16, 503–512. 10.1017/S0305000900010680 2808570

[B41] Singh L. Morgan J. L. Best C. T. (2002). Infants' listening preferences: baby talk or happy talk? Infancy 3, 365–394. 10.1207/S15327078IN0303_5 33451217

[B42] Soderstrom M. (2007). Beyond babytalk: re-evaluating the nature and content of speech input to preverbal infants. Dev. Rev. 27, 501–532. 10.1016/j.dr.2007.06.002

[B43] Sperry D. E. Sperry L. L. Miller P. J. (2019). Reexamining the verbal environments of children from different socioeconomic backgrounds. Child Dev. 90, 1303–1318. 10.1111/cdev.13072 29707767

[B44] Stross B. (1972). Verbal processes in Tzeltal speech socialization. Anthropological Linguist. 14, 1–13.

[B45] The ManyBabies Consortium (2020). Quantifying sources of variability in infancy research using the infant-directed-speech preference. Advan. Methods Pract. Psychol. Sci. 3, 24–52. 10.1177/2515245919900809

[B46] Weisleder A. Fernald A. (2013). Talking to children matters: early language experience strengthens processing and builds vocabulary. Psychol. Sci. 24, 2143–2152. 10.1177/0956797613488145 24022649 PMC5510534

[B47] Wickham H. (2016). ggplot2: Elegant Graphics for Data Analysis. New York, NY: Springer-Verlag.

[B48] Wittenburg P. Brugman H. Russel A. Klassmann A. Sloetjes H. (2006). ELAN: a professional framework for multimodality research, in Proceedings of the Fifth International Conference on Language Resources and Evaluation (Genoa), 1556–1559.

[B49] Zeidner M. (1983). ‘Kitchie-koo' in modern Hebrew: the sociology of Hebrew baby talk. Int. J. Soc. Lang. 41, 93–113. 10.1515/ijsl.1983.41.93

