# Supplementary material for: English-Speaking Adults' Labeling of Child- and Adult-Directed Speech Across Languages and Its Relationship to Perception of Affect
Source: Front Psychol. 2021 Sep 1;12:708887. doi: 10.3389/fpsyg.2021.708887 (PMC8440885; doi:10.3389/fpsyg.2021.708887)
Supplement: Supplementary file 1 [file Table_1.DOCX]

Supplemental Tables

Table 1

Full output for Experiment 1’s Binomial Mixed-effects Logistic Regression for accuracy.

|  | **accuracy** | | | |
| --- | --- | --- | --- | --- |
| *Predictors* | *Odds Ratios* | *std. Error* | *CI* | *p* |
| (Intercept) | 2.59 | 0.36 | 1.98 – 3.40 | **<0.001** |
| Addressee [cds] | 0.56 | 0.08 | 0.42 – 0.76 | **<0.001** |
| confidence | 1.33 | 0.05 | 1.23 – 1.44 | **<0.001** |
| Happy [Extremely Not] | 0.88 | 0.49 | 0.30 – 2.60 | 0.815 |
| Happy [Somewhat Not] | 0.82 | 0.12 | 0.62 – 1.09 | 0.167 |
| Happy [Somewhat] | 1.07 | 0.17 | 0.78 – 1.47 | 0.683 |
| Happy [Extremely] | 2.22 | 0.82 | 1.08 – 4.56 | **0.030** |
| Sooth [Extremely Not] | 0.93 | 0.33 | 0.47 – 1.85 | 0.835 |
| Sooth [Somewhat Not] | 1.03 | 0.18 | 0.73 – 1.45 | 0.875 |
| Sooth [Somewhat] | 0.33 | 0.06 | 0.23 – 0.47 | **<0.001** |
| Sooth [Extremely] | 0.21 | 0.13 | 0.06 – 0.68 | **0.010** |
| Love [Extremely Not] | 1.79 | 0.78 | 0.76 – 4.19 | 0.182 |
| Love [Somewhat Not] | 1.59 | 0.29 | 1.10 – 2.28 | **0.013** |
| Love [Somewhat] | 0.45 | 0.08 | 0.32 – 0.65 | **<0.001** |
| Love [Extremely] | 0.34 | 0.19 | 0.11 – 0.99 | **0.049** |
| Exaggerated [Extremely  Not] | 3.07 | 0.93 | 1.70 – 5.55 | **<0.001** |
| Exaggerated [Somewhat  Not] | 2.34 | 0.35 | 1.75 – 3.14 | **<0.001** |
| Exaggerated [Somewhat] | 0.66 | 0.10 | 0.49 – 0.90 | **0.009** |
| Exaggerated [Extremely] | 0.38 | 0.19 | 0.14 – 1.00 | **0.050** |
| Addressee [cds] * Happy  [Extremely Not] | 0.42 | 0.29 | 0.11 – 1.64 | 0.214 |
| Addressee [cds] * Happy  [Somewhat Not] | 0.79 | 0.18 | 0.51 – 1.24 | 0.307 |
| Addressee [cds] * Happy  [Somewhat] | 1.42 | 0.29 | 0.94 – 2.12 | 0.093 |
| Addressee [cds] * Happy  [Extremely] | 0.58 | 0.26 | 0.24 – 1.41 | 0.229 |
| Addressee [cds] * Sooth  [Extremely Not] | 0.45 | 0.23 | 0.17 – 1.23 | 0.118 |
| Addressee [cds] * Sooth  [Somewhat Not] | 0.75 | 0.18 | 0.46 – 1.21 | 0.232 |
| Addressee [cds] * Sooth  [Somewhat] | 4.78 | 1.14 | 3.00 – 7.62 | **<0.001** |
| Addressee [cds] * Sooth  [Extremely] | 19.96 | 14.25 | 4.92 – 80.90 | **<0.001** |
| Addressee [cds] * Love  [Extremely Not] | 0.60 | 0.40 | 0.17 – 2.18 | 0.441 |
| Addressee [cds] * Love  [Somewhat Not] | 0.42 | 0.11 | 0.25 – 0.71 | **0.001** |
| Addressee [cds] * Love  [Somewhat] | 4.10 | 0.97 | 2.58 – 6.52 | **<0.001** |
| Addressee [cds] * Love  [Extremely] | 7.91 | 5.08 | 2.25 – 27.85 | **0.001** |
| Addressee [cds] *  Exaggerated [Extremely  Not] | 0.11 | 0.05 | 0.05 – 0.25 | **<0.001** |
| Addressee [cds] *  Exaggerated [Somewhat  Not] | 0.19 | 0.04 | 0.13 – 0.29 | **<0.001** |
| Addressee [cds] *  Exaggerated [Somewhat] | 2.68 | 0.55 | 1.79 – 4.01 | **<0.001** |
| Addressee [cds] *  Exaggerated [Extremely] | 11.93 | 6.75 | 3.93 – 36.17 | **<0.001** |
| **Random Effects** | | | | |
| σ^2^ | 3.29 | | | |
| τ_00_ _recorded_childF_ | 0.06 | | | |
| ICC | 0.02 | | | |
| N _recorded_childF_ | 9 | | | |
| Observations | 6150 | | | |
| Marginal R^2^ / Conditional R^2^ | 0.420 / 0.430 | | | |
|  |  | | | |

|  |  |
| --- | --- |

Table 2

Full output for Experiment 1’s Binomial Mixed-effect Logistic Regression for Addressee (cds = 0, ads = 1).

|  | **Addressee** | | | |
| --- | --- | --- | --- | --- |
| *Predictors* | *Odds Ratios* | *std. Error* | *CI* | *p* |
| (Intercept) | 1.68 | 0.20 | 1.34 – 2.12 | **<0.001** |
| happy [Extremely Not] | 0.60 | 0.19 | 0.32 – 1.10 | 0.098 |
| happy [Somewhat Not] | 1.74 | 0.18 | 1.43 – 2.12 | **<0.001** |
| happy [Somewhat] | 0.50 | 0.04 | 0.42 – 0.59 | **<0.001** |
| happy [Extremely] | 0.71 | 0.13 | 0.50 – 1.01 | 0.056 |
| sooth [Extremely Not] | 1.36 | 0.30 | 0.88 – 2.11 | 0.161 |
| sooth [Somewhat Not] | 1.28 | 0.14 | 1.03 – 1.58 | **0.023** |
| sooth [Somewhat] | 0.56 | 0.06 | 0.45 – 0.69 | **<0.001** |
| sooth [Extremely] | 0.22 | 0.06 | 0.12 – 0.39 | **<0.001** |
| love [Extremely Not] | 1.02 | 0.27 | 0.60 – 1.72 | 0.947 |
| love [Somewhat Not] | 1.15 | 0.13 | 0.91 – 1.44 | 0.240 |
| love [Somewhat] | 0.49 | 0.05 | 0.40 – 0.60 | **<0.001** |
| love [Extremely] | 0.19 | 0.05 | 0.11 – 0.32 | **<0.001** |
| exaggerated [Extremely Not] | 2.33 | 0.40 | 1.66 – 3.26 | **<0.001** |
| exaggerated [Somewhat Not] | 1.49 | 0.14 | 1.25 – 1.78 | **<0.001** |
| exaggerated [Somewhat] | 0.32 | 0.03 | 0.27 – 0.38 | **<0.001** |
| exaggerated [Extremely] | 0.08 | 0.02 | 0.05 – 0.13 | **<0.001** |
| **Random Effects** | | | | |
| σ^2^ | 3.29 | | | |
| τ_00_ _recorded_childF_ | 0.08 | | | |
| ICC | 0.02 | | | |
| N _recorded_childF_ | 9 | | | |
| Observations | 6150 | | | |
| Marginal R^2^ / Conditional R^2^ | 0.585 / 0.595 | | | |

Table 3

Full output for Experiment 2b’s Binomial Mixed-effects Logistic Regression for accuracy.

|  | **accuracy** | | | |
| --- | --- | --- | --- | --- |
| *Predictors* | *Odds Ratios* | *std. Error* | *CI* | *p* |
| (Intercept) | 2.57 | 0.53 | 1.72 – 3.85 | **<0.001** |
| Addressee [cds] | 0.45 | 0.09 | 0.30 – 0.68 | **<0.001** |
| confidence.x | 0.87 | 0.03 | 0.80 – 0.93 | **<0.001** |
| Happy [Little] | 0.73 | 0.17 | 0.47 – 1.15 | 0.173 |
| Happy [Some] | 0.84 | 0.24 | 0.48 – 1.47 | 0.531 |
| Happy [More] | 1.68 | 0.70 | 0.75 – 3.79 | 0.209 |
| Happy [Extremely] | 0.36 | 0.27 | 0.08 – 1.55 | 0.169 |
| Sooth [Little] | 1.20 | 0.28 | 0.76 – 1.91 | 0.433 |
| Sooth [Some] | 0.75 | 0.22 | 0.42 – 1.33 | 0.326 |
| Sooth [More] | 0.64 | 0.30 | 0.25 – 1.61 | 0.343 |
| Sooth [Extremely] | 0.99 | 0.62 | 0.29 – 3.39 | 0.989 |
| Love [Little] | 0.84 | 0.20 | 0.53 – 1.33 | 0.446 |
| Love [Some] | 1.23 | 0.37 | 0.68 – 2.23 | 0.496 |
| Love [More] | 0.85 | 0.38 | 0.35 – 2.04 | 0.712 |
| Love [Extremely] | 1.15 | 0.77 | 0.31 – 4.26 | 0.836 |
| Excited [Little] | 0.91 | 0.21 | 0.58 – 1.42 | 0.666 |
| Excited [Some] | 0.50 | 0.13 | 0.30 – 0.82 | **0.006** |
| Excited [More] | 0.21 | 0.08 | 0.10 – 0.44 | **<0.001** |
| Excited [Extremely] | 0.37 | 0.21 | 0.13 – 1.10 | 0.073 |
| Addressee [cds] * Happy  [Little] | 1.21 | 0.32 | 0.73 – 2.02 | 0.454 |
| Addressee [cds] * Happy  [Some] | 1.35 | 0.43 | 0.72 – 2.53 | 0.357 |
| Addressee [cds] * Happy  [More] | 0.55 | 0.25 | 0.22 – 1.35 | 0.192 |
| Addressee [cds] * Happy  [Extremely] | 4.97 | 4.08 | 0.99 – 24.83 | 0.051 |
| Addressee [cds] * Sooth  [Little] | 0.88 | 0.23 | 0.52 – 1.47 | 0.621 |
| Addressee [cds] * Sooth  [Some] | 1.05 | 0.34 | 0.55 – 2.00 | 0.889 |
| Addressee [cds] * Sooth  [More] | 1.70 | 0.88 | 0.62 – 4.66 | 0.303 |
| Addressee [cds] * Sooth  [Extremely] | 1.04 | 0.73 | 0.26 – 4.13 | 0.960 |
| Addressee [cds] * Love  [Little] | 1.04 | 0.28 | 0.62 – 1.75 | 0.882 |
| Addressee [cds] * Love  [Some] | 0.86 | 0.29 | 0.44 – 1.66 | 0.652 |
| Addressee [cds] * Love  [More] | 1.72 | 0.86 | 0.64 – 4.58 | 0.280 |
| Addressee [cds] * Love  [Extremely] | 0.88 | 0.65 | 0.21 – 3.70 | 0.863 |
| Addressee [cds] * Excited  [Little] | 1.36 | 0.35 | 0.82 – 2.25 | 0.233 |
| Addressee [cds] * Excited  [Some] | 3.12 | 0.91 | 1.76 – 5.51 | **<0.001** |
| Addressee [cds] * Excited  [More] | 9.44 | 4.12 | 4.02 – 22.19 | **<0.001** |
| Addressee [cds] * Excited  [Extremely] | 5.52 | 3.43 | 1.64 – 18.62 | **0.006** |
| **Random Effects** | | | | |
| σ^2^ | 3.29 | | | |
| τ_00_ _recording_ | 0.00 | | | |
| ICC | 0.00 | | | |
| N _recording_ | 9 | | | |
| Observations | 2631 | | | |
| Marginal R^2^ / Conditional R^2^ | 0.064 / 0.064 | | | |

Table 4

|  | **Addressee.num** | | | |
| --- | --- | --- | --- | --- |
| *Predictors* | *Odds Ratios* | *std. Error* | *CI* | *p* |
| (Intercept) | 0.21 | 0.14 | 0.06 – 0.79 | **0.021** |
| Happy [Little] | 0.98 | 0.14 | 0.74 – 1.31 | 0.902 |
| Happy [Some] | 0.86 | 0.15 | 0.60 – 1.21 | 0.379 |
| Happy [More] | 0.76 | 0.19 | 0.47 – 1.23 | 0.258 |
| Happy [Extremely] | 0.85 | 0.32 | 0.40 – 1.79 | 0.668 |
| Sooth [Little] | 1.05 | 0.15 | 0.79 – 1.39 | 0.750 |
| Sooth [Some] | 1.19 | 0.21 | 0.84 – 1.67 | 0.334 |
| Sooth [More] | 1.03 | 0.27 | 0.61 – 1.71 | 0.923 |
| Sooth [Extremely] | 2.71 | 0.95 | 1.36 – 5.38 | **0.005** |
| Love [Little] | 0.91 | 0.13 | 0.68 – 1.21 | 0.517 |
| Love [Some] | 1.01 | 0.18 | 0.71 – 1.43 | 0.962 |
| Love [More] | 1.05 | 0.27 | 0.64 – 1.75 | 0.837 |
| Love [Extremely] | 0.72 | 0.27 | 0.35 – 1.48 | 0.378 |
| Exaggerated [Little] | 1.08 | 0.15 | 0.82 – 1.43 | 0.587 |
| Exaggerated [Some] | 1.03 | 0.17 | 0.74 – 1.42 | 0.882 |
| Exaggerated [More] | 1.08 | 0.25 | 0.69 – 1.69 | 0.733 |
| Exaggerated [Extremely] | 0.70 | 0.23 | 0.36 – 1.35 | 0.286 |
| **Random Effects** | | | | |
| σ^2^ | 3.29 | | | |
| τ_00_ _recording_ | 3.75 | | | |
| ICC | 0.53 | | | |
| N _recording_ | 9 | | | |
| Observations | 2631 | | | |
| Marginal R^2^ / Conditional R^2^ | 0.006 / 0.535 | | | |
